# Typing of *Salmonella* Typhi strains isolated from Egypt by RAPD PCR

**DOI:** 10.1007/s13205-011-0022-8

**Published:** 2011-08-30

**Authors:** Noha A. Rezk, Hoda Mansour, Nahed H. Ghoneim, Mahmoud M. Rifaat

**Affiliations:** 1 R&D Department, Holding Company for Biological Products and Vaccines (VACSERA), Giza, 12511 Egypt; 2 Naval Medical Research Unit No. 3 (NAMRU-3), Cairo, 11591 Egypt; 3 Department of Zoonosis, Faculty of Veterinary Medicine, Cairo University, Giza, 12511 Egypt; 4 Department of Biotechnology, Faculty of Science, Taif University, Taif, 21974 Kingdom of Saudi Arabia

**Keywords:** RAPD profiles, RAPD types, Discrimination Index, Cluster analysis, Principal component analysis, Biclustering

## Abstract

PCR-based fingerprinting using random amplified polymorphic DNA (RAPD) has been used widely for genome identification. In this study, 13 *Salmonella* Typhi strains were isolated from typhoid patients from Aswan, Cairo, Fayoum, and Monofya Governorates of Egypt. The isolates, along with three reference strains, i.e., O901, H901, and Ty2 were subjected to whole genome typing by RAPD PCR. Three RAPD-PCR 10-mer primers generated a total of 85 RAPD bands (81 polymorphic bands), 12 distinct PCR profiles, and proved to be useful for discriminating the isolates and strains studied. Interestingly, the B_1_ and C_1_ PCR profile were found only in Cairo and Monofya, respectively; and some PCR types appeared only in certain Governorates of Egypt. By combining the profiles obtained with the primer trio used in this study, an excellent discrimination index (*D*) of 0.942 was reached. Pairwise comparisons of Jaccard’s similarity coefficients calculated among the 12 PCR types identified three major clusters; i.e., O901 branch and Ty2 and H901 sub-branches. Principal component analysis adequately resolved each of these three major clusters. Three principal components accounted for about 72% of the variation, with the first two components accounting for about 62% of the total variance among the genotypes studied. Biclustering improved the display of groups of RAPD amplicons (markers) that cluster similarly across the genomes and could delineate features pertaining to genome structure. In conclusion, RAPD PCR provided a fast method with high potentials in surveillance and epidemiological investigations of *Salmonella* Typhi infections.

## Introduction

Typhoid fever is a systemic infection with the bacterium *Salmonella enterica* serotype Typhi. This fever is an important cause of illness and death with a global occurrence of 21.6 million infections and about 200,000 deaths from typhoid fever per year (Bhutta 2006). In Egypt, the population-based incidence of typhoid fever in Fayoum Governorate was 59/100,000 persons/year. A concerning prevalence of multidrug-resistant *Salmonella* Typhi (29%) was reported (Srinkantiah et al. 2006).

*Salmonella* Typhi has evolved a genetic mechanism for the expression of virulence genes located at pathogenicity islands in the bacterial genome. The size, structure, function, and distribution of these islands in the genomes of *Salmonella* subspecies and serovars can be markedly different (Hensel 2004; Aguirre et al. 2006; Akiba et al. 2006). As revealed by sequencing and microarray analyses, the genome of *Salmonella* Typhi also accumulated many pseudogenes (McClelland et al. 2004).

The success of epidemiological surveillance studies of *Salmonella* is related to the typing procedures applied to differentiate the genotypes. Several typing methods for *Salmonella* have been described for epidemiological and phylogenetic purposes (Ruiz et al. 2003; Lim et al. 2005; Araque 2009; Nath et al. 2010). The DNA-based typing methods are becoming increasingly useful for performing epidemiological surveys of pathogenic bacteria. Randomly amplified polymorphic DNA (RAPD) analysis, also known as arbitrarily primed-polymerase chain reaction (AP-PCR), is based on the presence of primer binding sites in the genome close enough to permit PCR amplification using a single primer with arbitrary nucleotide sequence at low annealing temperature. RAPD PCR, among other genetic typing procedures, has been shown to be useful tool to trace *Salmonella* epidemiologically and to distinguish *Salmonella* strains from different geographical origins. RAPD typing was useful for epidemiological typing of *Salmonella* isolates from human outbreaks and from avian sources and for complementing serotyping and phage typing methods (Soto et al. 1999, 2000; De Cesare et al. 2001; Lim et al. 2005; Quintaes et al. 2004; Smith et al. 2011).

The objective of this study is to develop a simple, easy to interpret, and low cost RAPD-based method, for typing *Salmonella* Typhi isolated from Egyptian patients suffering from typhoid fever. As RAPD PCR detects sequence diversity of total DNA, it is expected to display a good degree of sequence divergence. The method does not require any specific knowledge of the DNA sequence of the target organisms. This makes it a flexible tool that has great power and general applicability. Moreover, the method is more rapid and less technically demanding than most other molecular typing methods.

## Materials and methods

### Samples

Anti-coagulated whole peripheral blood (WPB) samples were collected from 300 patients suffering from prolonged fever and admitted to the Abbassia Fever Hospital (Cairo), Fayoum General Hospital (Fayoum), Aswan General Hospital (Aswan), and Shebein General Hospital (Monofya). *Salmonella enterica* serotype Typhi (*Salmonella* Typhi) was isolated by blood culture from 13 (4.3%) patients. Three reference *Salmonella* Typhi strains; i.e., TY2, O901, and H901 as well as *E. coli* JM109 strain (non-*Salmonella* control) were also included in this study (Table 1).

**Table 1:** Isolates and reference strains of *Salmonella* Typhi included in this study

Isolate/strain	Area	Sample	Isolate/strain	Area	Sample
H901	–	Reference Strain	C-Han	Cairo	Blood (WPB)
O901	–	Reference Strain	C-Nag	Cairo	Blood (WPB)
Ty2	–	Reference Strain	C-QC1	Cairo	Blood (WPB)
A-Ash	Aswan	Blood (WPB)	F-33	Fayoum	Blood (WPB)
A-215	Aswan	Blood (WPB)	F-37	Fayoum	Blood (WPB)
C-160	Cairo	Blood (WPB)	F-Kha	Fayoum	Blood (WPB)
C-867	Cairo	Blood (WPB)	S-43	Shebein (Monofya)	Blood (WPB)
C-Bat	Cairo	Blood (WPB)	S-Mah	Shebein (Monofya)	Blood (WPB)

### Materials

Bacterial identification kit was obtained from Bio-merieux Vitek Inc., (Hazelwood, Missouri, USA). Selective enrichment media (Selenite “F” enrichment broth) and plating media (MacConkey lactose bile salt agar and *Salmonella*–*Shigella* agar) were obtained from Becton, Dickinson Microbiology Systems (NJ, USA). *Salmonella* O and H antisera employed in the identification of somatic and flagellar antigens were obtained from SA Scientific Inc. (San Antonio, TX). Molecular biology grade reagents were purchased from Roche (Roche Diagnostics GmbH, Germany), New England Biolabs Inc. (MA, USA), FMC Bioproducts (Rockland, Maine, USA), and GeneCraft (GeneCraft GmbH, Germany).

### Primers

Oligonucleotides were commercially synthesized by Operon (Operon, A Qiagen Company, Qiagen GmbH, Germany). Nine 10-mer primers were used for RAPD-PCR typing (Table 2). All primers used were resuspended in TE buffer, stored at −20 °C, and 10 μM (10 pm/μl) working solutions were prepared to be used in PCR.

**Table 2:** Oligonucleotides used as primers in RAPD PCR

Primer	Sequence (5′–3′)	GC (%)	*T*_m_ (ºC)	Mol Wt (Da)
B02	TGA TCC CTG G	60	32.2	3,019
A10	GTG ATC GCA G	60	33.1	3,068
E10	CAC CAG GTG A	60	33.4	3,037
A02	TGC CGA GCT G	70	40.7	3,044
A09	GGG TAA CGC C	70	37.4	3,053
E18	AGG TGA CCG T	60	36.2	3,068
A13	CAG CAC CCA C	70	37.7	2,942
C02	GTG AGG CGT C	70	37.6	3,084
D05	TGA GCG GAC A	60	37.1	3,077

### Preparation of genomic DNA

Bacterial genomic DNA was prepared according to Sambrook et al. (2001). The bacteria were grown overnight at 37 °C in Luria–Bertani (LB) broth prior to DNA preparation. Lysozyme was used at a final concentration of 3 mg/ml. Sodium dodecyl sulfate (SDS) was added to final concentration of 1%, and proteinase K was used at a final concentration of 50 μg/ml. The prepared DNA was further purified using the High Pure PCR template preparation Kit (Roche Diagnostics GmbH, Germany) according to the protocol provided by the manufacturer. The quality of *Salmonella* Typhi DNA prepared in this study was verified by PCR amplification of the16S rDNA from the bacterial genome.

### RAPD PCR

The reaction mixture (25 μl) contained 10 mM Tris–HCI pH 7.5, 50 mM KCI, 1.5 mM MgCl_2_, 0.5 mM spermidine, 0.1 mM dNTPs, 15 pmol of the RAPD primer, 20 ng genomic DNA, and 0.8 U of *Taq* DNA polymerase. Amplification was carried out in a heated-lid Biometra Thermal Cycler (Biometra GmbH, Germany) for 40 cycles, each consisting of a denaturing step of 1 min at 94 °C, followed by annealing step of 1 min at 36 °C and an extension step of 2 min at 72 °C. The last cycle was followed by 5 min of long extension at 72 °C. The amplification products were separated by gel electrophoresis in 2.2% agarose (SeaKem LE agarose; FMC Bioproducts, Rockland, Maine, USA) in 45 mM Tris–borate, 1 mM EDTA buffer (pH = 8.0), containing ethidium bromide at 0.5 μg/ ml at a constant voltage of 5 V/cm. The gels were photographed under UV transillumination using a digital camera inside a gel documentation system (Alpha Innotech, CA, USA).

### Analysis of RAPD PCR Data

Gel images were analyzed for genetic similarity among isolates using the AlphaEase Software (Alpha Innotech, CA, USA). RAPD bands were scored as discrete variables, using “1” to indicate presence and “0” to indicate the absence of a band in the profile. The PCR profiles are defined by the pattern of presence or absence of bands on the gel. Each PCR profile was labeled with an alphabetical letter followed by a numeral subscript that identifies the primer used to generate the PCR profile. Each amplicon was assigned a name that begins with the letter P followed by a number that indicates the RAPD primer used, and a 2-digit number that identifies the band position. The discrimination index (*D*) was calculated for each primer by using Simpson’s index of diversity as described by Hunter and Gaston (1988) as follows:Where *N* total number of strains in the population studied, *S* total number of RAPD types, *n*_*j*_ number of strains belonging to the *j*th type, *n*_*j*_*/N t*he probability that a single strain sampled at ransom will belong to the *j*th type, and *n*_*j*_*(n*_*j* _*−* 1)/*N*(*N* − 1) the probability that two strains sampled consecutively will belong to the same *j*th type.

The similarities between DNA fingerprints were calculated with the band-matching Jaccard’s coefficient that ranges from 0 to 1.0, where 1.0 represents 100% identity (presence and position) for all bands in the two PCR fingerprints being compared. A pairwise similarity (or distance) matrix was developed and cluster analysis was performed using the Unweighted Pair Group Method with Arithmetic averages (UPGMA) method. Principal component analysis (PCA), a mathematical procedure that uses orthogonal linear transformation, was used to recognize patterns in the RAPD-generated markers and to highlight the relationships between the genotypes examined. PCA and biclustering, i.e., clustering of RAPD types and RAPD amplicons were performed by using the Cluster and TreeView program of Stanford University, USA (Eisen et al. 1998).

## Results and discussion

The *Salmonella* Typhi isolates as well as the three reference strains studied were found to give reproducible RAPD profiles from the same or from newly-extracted genomic DNA purified as described under “Materials and methods” and stored at −20 °C. The reproducibility of RAPD profiling, i.e., the ability to amplify the same RAPD profile and to observe the same polymorphism, was tested in at least three replicates.

**Fig. 1:**
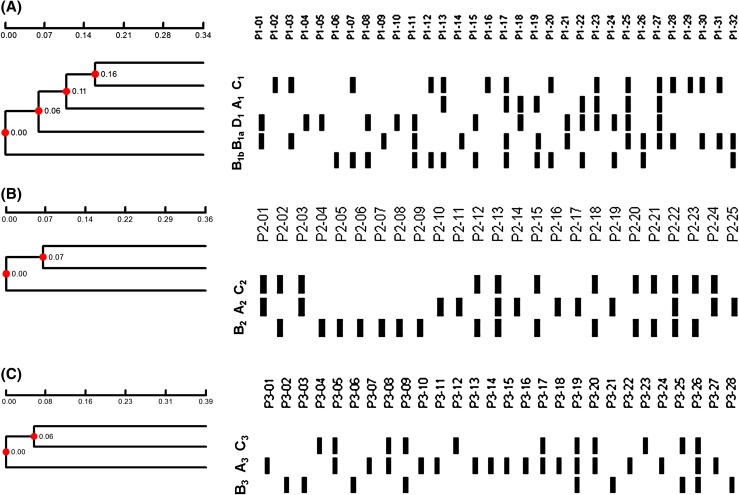
Clustering of *Salmonella* Typhi RAPD profiles based on Jaccard’s coefficient and the UPGMA method. Band matching was based on the adjusted *R*_f_ values (AlphaEase FC Software, Alpha Innotech, CA, USA). The PCR profiles were labeled with alphabetical letters (*A*, *B*, *C*, and *D*) followed by the numerals (1, or 2, or 3) as subscripts. The numeral subscripts were respectively assigned to the three primers, B02, E10, and E18. **a** Profiles A_1_, B_1a_, B_1b_, C_1_, and D_1_ generated by RAPD primer B02. **b** Profiles A2, B2, and C2 generated by RAPD primer E10. **c** Profiles A_3_, B_3_, and C_3_ generated by RAPD primer E18. Reference profiles were A_1_, A_2_, and A_3_. Each amplicon was assigned a name that begins with the letter P followed by a number (1, or 2, or 3) that indicates the primer used (B02, E10, and E18, respectively), and a 2-digit number that identifies the band position. The direction of electrophoretic migration was from left to right, and band numbering starts from the large amplicons in each profile

Out of the nine primers tested, only three primers, i.e., B02, E10, and E18 were suitable for discrimination (Table 2). Each of the three primers produced at least three distinct PCR profiles from the DNA preparations of *Salmonella* Typhi isolates and strains studied (Fig. 1), as well as, a distinct profile from *E. coli* (non-*Salmonella* control). The remaining primers produced less than 3 distinct PCR profiles. The PCR profiles generated by primers B02, E10, and E18 included 8–18 amplicons that ranged in size from 200–1,500 bp. These three primers were, therefore, used to reproducibly amplify random fragments of DNA from *Salmonella* Typhi genome.

A total of 85 RAPD bands (81 polymorphic bands) were scored. The four monomorphic bands were produced by RAPD primer E10 (P2-13 and P2-22) and primer E18 (P3-19 and P3-26). The numbers of RAPD bands resolved were 32 (all polymorphic, 39.5%), 25 (23 polymorphic, 28.4%), and 28 (26 polymorphic, 32.1%), for the primers B02, E10, and E18, respectively. On the average, 27 polymorphic RAPD markers were produced per RAPD primer from the studied genomes. The numbers of PCR profiles amplified by the three primers were 5, 3, and 3, respectively (Table 4).

The RAPD primer B02 showed the highest discriminatory index (*D* = 0.675) and identified five distinct PCR profiles (A_1_, B_1a_, B_1b_, C_1_, and D_1_) from 16 *Salmonella* Typhi samples examined (Fig. 1). The B_1_ and C_1_ PCR profiles were only found in the samples collected from Cairo and Fayoum, respectively (Table 3). This RAPD primer was useful for distinguishing the reference strain O901 (A_1_ PCR profile) from the rest of the *Salmonella* Typhi isolates and strains studied. The other two reference strains (Ty2 and H901) were also distinguishable by this primer and belonged to different PCR profiles (B_1a_ and D_1_ profiles, respectively). Thus, the three reference strains yielded different band profiles with this primer. This observation agrees with those of McKenna et al. (1995) on the differentiation of Ty21a vaccine strain from the rest of *Salmonella* Typhi isolates using a single arbitrary primer, and those reported by Gürakan et al. (2008) on the use of a single RAPD primer as a serotype-specific marker for *Salmonella* Typhimurium.

**Table 3:** RAPD PCR profiles and types generated from *Salmonella* Typhi isolates and strains using the three RAPD primers

Isolate/strain	Profiles amplified by RAPD primer			PCR type
	B02	E10	E18	
O901	A_1_	A_2_	C_3_	1
Ty2	B_1a_	B_2_	A_3_	2
C-867	B_1b_	B_2_	B_3_	3
C-160	B_1a_	B_2_	C_3_	4
C-Bat	B_1b_	C_2_	C_3_	5
S-43	C_1_	B_2_	B_3_	6
S-Mah	C_1_	B_2_	C_3_	7
A-215	D_1_	A_2_	C_3_	8
F-33				
H901	D_1_	B_2_	A_3_	9
F-37	D_1_	B_2_	B_3_	10
C-Han	D_1_	B_2_	C_3_	11
F-Kha				
C-QC1				
C-Nag				
A-Ash	D_1_	C_2_	A_3_	12

Governorates: *A* Aswan, *C* Cairo, *F* Fayoum, and *S* Monofya

The RAPD primer E10 had the lowest discriminatory index (*D* = 0.508) and identified only three distinct PCR profiles (A_2_, B_2_, and C_2_) from the isolates and strains examined. Two RAPD amplicons (P2-13 and P2-22) were common among these three PCR profiles (Fig. 1), while the other amplicons were useful for assigning *Salmonella* Typhi into the different profiles. The reference strains O901 and H901 had different PCR profiles with this primer. The RAPD primer E18 had an intermediate discriminatory index (*D* = 0.575) and identified three distinct PCR profiles (A_3_, B_3_, and C_3_) from the isolates and strains examined. Two RAPD amplicons (P3-19 and P3-26) were monomorphic among the three PCR profiles produced by this primer (Fig. 1). The reference strains H901 and Ty2 were indistinguishable by the two primers E10 or E18. Table (4) shows that the most frequent PCR profiles generated by primers B02, E10, and E18 were D1, B_2_, and C_3_, respectively. The D_1_ and B_2_ PCR profiles were found in three Governorates, while C_3_ PCR profile was found in the four Governorates.

**Table 4:** RAPD primers, profiles, and types of *Salmonella* Typhi isolates and strains examined

	Primer			
	B02	E10	E18	Combined
Total number of amplicons	32	25	28	85
Polymorphic amplicons	32	23	26	81
% of all polymorphic amplicons	(39.5%)	(28.4%)	(32.1%)	(100%)
Monomorphic amplicons	0	2	2	4
Discrimination index (*D*)	0.675	0.508	0.575	0.942
Number of PCR profiles/types	5	3	3	12
Most frequent PCR profile/type	D_1_ (9/16)	B_2_ (11/16)	C_3_ (10/16)	11 (4/16)
Distribution				
Cairo	√	√	√	√
Monofya		√	√	
Fayoum	√	√	√	√
Aswan	√		√	
Most similar profile(s)/type(s)	A_1_ and C_1_	A_2_ and C_2_	A_3_ and C_3_	9 and 12
Profile(s)/type(s) with the highest % of polymorphic bands	B_1a_ (41%) C_1_ (41%)	B_2_ (52%)	A_3_ (57%)	2 (54.3%)
Profile(s)/type(s) with the lowest % of polymorphic bands	A_1_ (19%)	A_2_ (40%) C_2_ (40%)	B_3_ (25%)	1 (33.3%)

In this study, the three RAPD primers used were able to generate 12 distinct PCR types by combining the profiles obtained with the three primers (Table 3). The most frequent PCR type was type 11 (4 isolates). A discrimination index (*D*) of 0.942 was achieved for the combined RAPD typing used in this study (Table 4). Lim et al. (2005) reported that a combination of two different RAPDs or a combination of RAPD and ERIC was better than the other combinations for the differentiation of field-isolated *Salmonella* strains and epidemiological studies. Combining the PCR types identified by the RAPD typing and phage typing or combining RAPD and PFGE increased the discrimination capacity (Soto et al. 1999; Delgado Ronda et al. 2006). The combination of RAPD typing with antibiotic susceptibility testing was a reliable discriminatory approach to differentiate *Salmonella* for epidemiologic purposes in Iran (Madadgar et al. 2008).

**Fig. 2:**
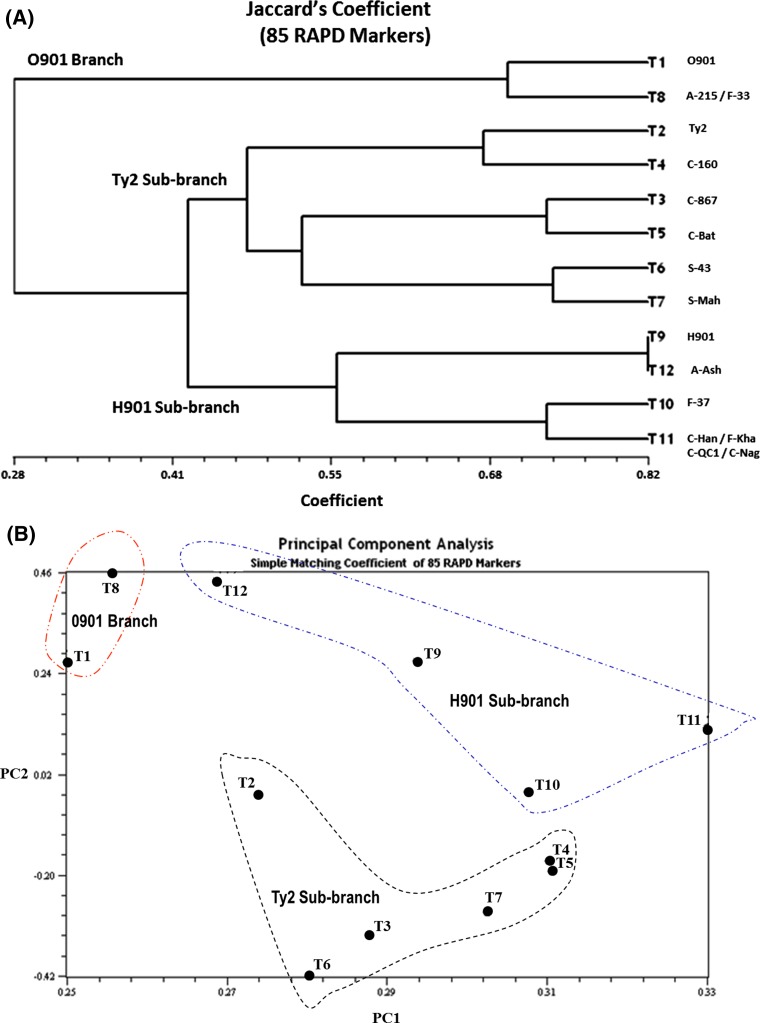
**a** The dendrogram constructed from the pairwise comparisons of Jaccard’s similarity coefficients and calculated based on the 85 RAPD markers. A major branch (O901 branch) was identified, as well as, two sub-branches (Ty2 and H901 sub-branches). **b** Results of principal component analysis (PCA) showing the two-dimensional (PC1 and PC2) plot. The first two principal components (PC1 and PC2) resolved the major branch (O901 branch) and each of the two sub-branches (Ty2 and H901). *T1*–*T12* indicate the twelve RAPD PCR types

 Jaccard’s similarity coefficients, based on the 85 RAPD markers, identified three major clusters, i.e., O901 branch and Ty2 and H901 sub-branches (Fig. 2a).

The O901 branch had the reference strain O901 (PCR type 1) along with PCR type 8. The Ty2 sub-branch contained the reference strain Ty2 along with five PCR types (i.e., Types 3–7), while the H901 sub-branch clustered the reference strain H901 (Type 9) along with three PCR types (Types 10–12). The reference strain H901 (Type 9) and the isolate A-Ash (Type 12) clustered closely in this sub-branch, with a Jaccard’s similarity coefficient of 0.82, indicating a close genetic similarity.

The results of principal component analysis (PCA), performed on the 85 RAPD markers, showed that the first (PC1), second (PC2), and third (PC3) principal components explained 48.52, 13.27, and 10.77% of the total variation in RAPD data, respectively. The first two components accounted for about 62% of the total variance, and the RAPD-PCR data were represented adequately by three principal components accounting for about 72% of the total variation. The results of PCA analysis were in considerable agreement with the overall representation of the genomes revealed by pairwise comparisons of Jaccard’s similarity coefficients. In the two-dimensional PCA plot, nearly all of the RAPD PCR types were clearly distinct from each other, and the major branch (O901 branch) and the two sub-branches (Ty2 and H901) could be resolved (Fig. 2b). Thus, the results of cluster analysis were supported by those of principal component analysis (PCA).

**Fig. 3:**
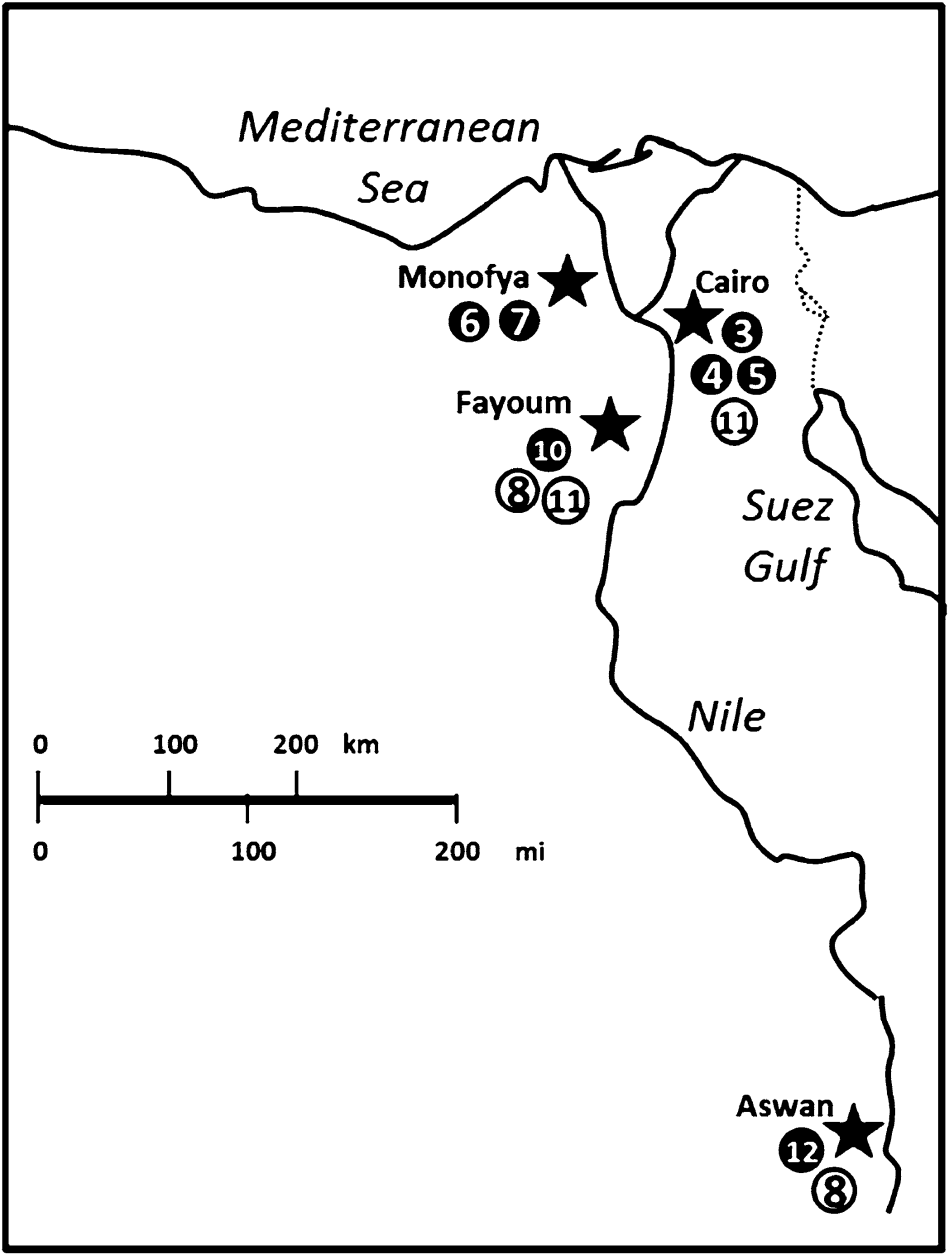
Locations of *Salmonella* Typhi RAPD types identified in this study. The RAPD types representing the reference strains O901, Ty2, and H901 are not shown. *Encircled numbers* indicate the RAPD types. A *black-filled circle* indicates that this RAPD type appeared only in this location

Apart from the most frequent PCR types (types 8 and 11), different PCR types were found in Cairo, Monofya, Fayoum, and Aswan (indicated by black-filled circles in Fig. 3). The most frequent PCR types (types 8 and 11) were not among the most similar PCR types (types 9 and 12). Likewise, the most frequent profiles generated by primers B02 and E10 were not among the most similar profiles generated by these primers (Table 4). The data, therefore, indicated that there is no close relationship between the most frequent PCR types observed. This supports the conclusion that the isolates of *Salmonella* Typhi had a considerable degree of genetic heterogeneity. However, due to the small sample size, the relationship between profiles and localities could not be established significantly. Previous studies on molecular typing and phylo-geographical distribution of *Salmonella* Typhi showed that different strains could be in circulation in endemic areas and outbreaks are related to only a few strains (Soto et al. 2000; Baker et al. 2010). Among *Salmonella* isolated in three provinces in the midwest of Spain, additional smaller clonal lines coexisted within every area. Most cases of infections are caused by the epidemic spread of a clone or, at least, by a group of strains that are genetically very close. Emergence and spread of *Salmonella* resulted mainly from the most frequent lineages (Soto et al. 2000; Delgado Ronda et al. 2006).

**Fig. 4:**
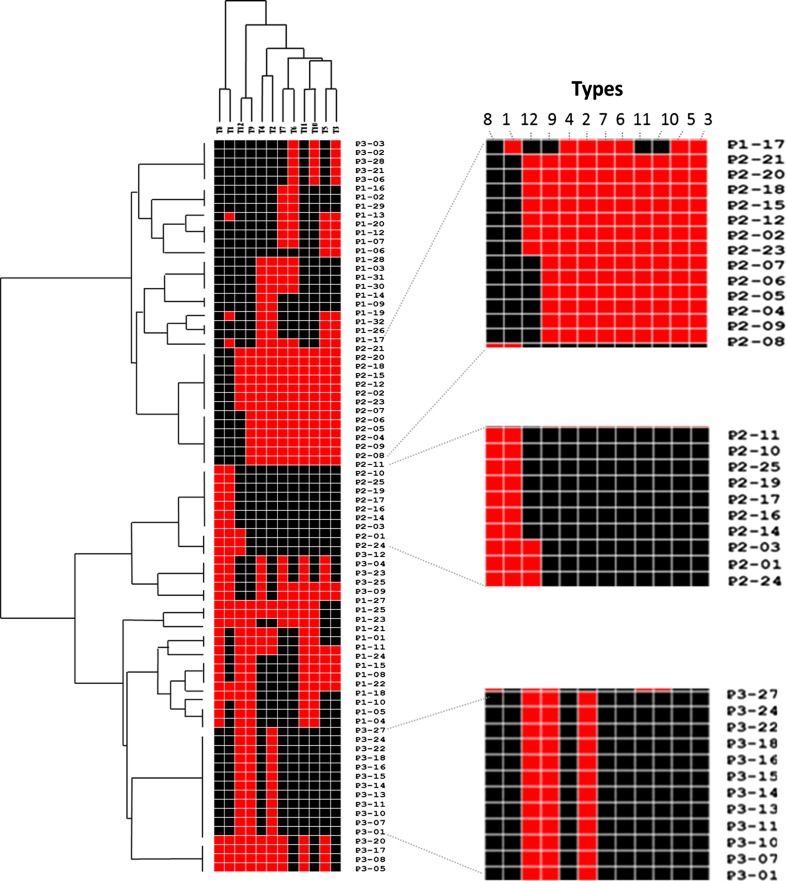
Biclustering of RAPD PCR types (genomes) and RAPD PCR amplicons (markers). Each RAPD marker is represented by a *single row* and each PCR type is represented by a *single column*. The *red color* indicates the presence of the RAPD-PCR amplicon in the bacterial genome, while the *black color* indicates the absence of the amplicon in the bacterial genome. The two dendrograms were generated using the Cluster and TreeView program

The biclustering algorithm was used to reorder the RAPD PCR types (genomes) and the RAPD PCR amplicons (markers) in order to optimize grouping and to visualize the data as a heat map. The biclustering indicated that certain RAPD amplicons (markers) cluster similarly across the PCR types (genomes). For example, RAPD amplicons P2-21 to P2-23 were absent from PCR types 1 and 8, while RAPD amplicons P2-07 to P2-08 were absent from PCR types 1, 8, and 12 (Fig. 4). On the other hand, RAPD amplicons P2-11 to P2-14 were only present in PCR types 1 and 8, while RAPD amplicons P2-03 to P2-24 were only present in PCR types 1, 8, and 12. RAPD amplicons P3-27 to P3-01 were present only in PCR types 2, 9, and 12.

It is conceivable that several features pertaining to genome architecture in *Salmonella* could be revealed by biclustering. The genome of *Salmonella* Typhi is characterized by the presence of a large number of genetic elements (*Salmonella* Pathogenicity Islands or SPI) that can be acquired by horizontal transmission to allow bacteria to rapidly gain complex virulence functions and to enable the emergence of new antibiotic-resistant epidemic strains. About 7.8% of the genome of *Salmonella* Typhi consists of pathogenicity islands and several common sequence motifs were identified between SPIs (Hensel 2004). These features, however, may be elucidated further using typing methods that use consensus oligonucleotides to reveal the presence of dispersed repetitive DNA sequences.

Due to the polymorphism inherent in the sequence, as well as, the short length of the RAPD primers used, the RAPD typing method resulted in a clustering of isolates into highly discriminating genetic trees. Although relatively few samples were used in this study, the data suggest that RAPD typing is discriminatory, it is easy to interpret and constitutes a low cost method to type the various strains of *Salmonella* Typhi. The high discriminatory power (*D* = 0.942) of the RAPD typing method used in this study revealed the robustness of the method and promises a high potential as a molecular typing method in surveillance and local outbreak investigations of *Salmonella* Typhi infections.
